# Outcomes of Revascularization and Factors Associated With Major Amputation in Patients With Lower Limb Arterial Injury: A Single-Center Retrospective Analysis

**DOI:** 10.7759/cureus.17290

**Published:** 2021-08-18

**Authors:** Ghulam Ali, Muhammad Fahad Berlas, Najam U Din, Khalil Ur Rehman, Waryam Muhammad Saleh, Syed Arsalan Ahmed Naqvi

**Affiliations:** 1 Vascular Surgery, Shaheed Mohtarma Benazir Bhutto Institute of Trauma, Karachi, PAK; 2 Internal Medicine, Dow University of Health Sciences, Karachi, PAK

**Keywords:** lower limb amputation, amputation, peripheral revascularization, limb salvage procedures, complex lower limb trauma, arterial injury, failed revascularization

## Abstract

Objective

To identify the amputation rates and causative factors for failed revascularization leading to amputation in patients undergoing primary limb salvage procedures for lower-extremity vascular injuries.

Methods

This retrospective study was conducted at the vascular surgery department, Shaheed Mohtarma Benazir Bhutto (SMBB) Institute of Trauma, Karachi, Pakistan. The data were collected from hospital record using the non-probability sampling technique. Patients aged 17-70 years, undergoing primary revascularization during April 2016 to March 2021, were included in the study. Patients with crush injuries/non-salvageable limbs underwent primary amputation, isolated deep femoral artery or crural arteries (non-limb threatening) injuries, and non-traumatic injuries like intravenous drug-induced or iatrogenic injuries were excluded. The data analysis is done using SPSS Version 20.0 (IBM Corp., Armonk, NY, USA). A P-value of <0.05 was considered as significant.

Results

This study includes 56 patients of mean age 30.82 ± 9.29 years with male gender four times more affected than their counterpart. About 32% of patients were smokers, while 58% of patients had no co-morbidities. All patients presented with a mean time of 7.66 ± 1.69 hours of injury with an average of 1.14 arterial segments involved. The most frequent artery involved was popliteal artery (both above and below the knee), followed by superficial femoral artery injury constituting 50% and 26%, respectively, with arterial laceration and transection being common findings on exploration. Out of 56 patients, 27 (48.2%) had open fractures, 21 (37.5% ) closed fractures, and eight patients (14.3 % ) presented with dislocation as associated injuries. Following the procedure, secondary amputation was recorded in 18 (32.1%) patients. Thrombosis and infection were the leading causes of revascularization failure. Type of injury, segment of arterial injury, and associated bony injuries were associated with limb amputation.

Conclusion

Type and site of injury along with concomitant bony injuries are associated with major amputations after revascularization in lower-extremity arterial injuries.

## Introduction

In this contemporary era, the most common cause of morbidity and mortality is from traumatic injuries affecting the young to middle age population, posing a huge burden on any nation’s overall economy. Vascular injuries are one of the devastating injuries leading to limb or life loss. No precise data are available to suggest the overall incidence of traumatic vascular injuries involving lower limbs in Pakistan; however, one study revealed that popliteal artery injuries are the second commonly affected lower limb vascular injuries [1].

Extremity vascular injuries are relatively rare (4%-6%) and associated with overwhelming soft tissue and bony injuries [2]. Popliteal artery is injured in about 0.2% of all traumatic peripheral injuries and is almost always associated with bony and soft tissue injuries, and it leads to poor functional outcomes and significantly higher amputation rates [2-4]. The main objective of surgical intervention is to salvage the injured limb by revascularization and achieve a functional limb. However, sometimes this intervention can result in devastating complications such as reperfusion injury and compartment syndrome, compared to an early amputation and rehabilitation [5,6]; hence, proper patient selection and timely intervention is the key to achieve desirable outcomes in terms of functional limb.

The main aim of identifying patients who will benefit from salvage intervention (revascularization) is paramount to obtain the optimum outcome [7]. Early revascularization (within 6 hours of injury) is the key to prevent neuromuscular tissue ischemia and, hence, improved functioning outcomes [8]. The most common causes of failed revascularization are thrombosis, infection, and advanced necrosis regardless of patent repair leading to eventual limb loss [5,9]. Surgeons usually have limited decision-making expertise, and assessment tools are not supportive in estimating revascularization outcomes, leaving patients to suffer from consequences [10]. One such supportive tool in predicting limb salvage is the Mangled Extremity Severity Score (MESS); however, despite good MESS score (<7), multiple failed revascularization attempts eventually lead to amputation of the affected limb [10]. Our country has very few dedicated public and private trauma centers where vascular interventions are routine in traumatic peripheral vascular injuries. The scarcity of resources raises the question of effective utilization of efforts and scrutinizes patients who will benefit from the intervention. This research is aimed to determine the frequency of amputations and to identify factors associated with major amputation following a revascularization attempt during the same in-hospital stay in patients with lower limb traumatic vascular injuries.

## Materials and methods

This observational, retrospective research was conducted from April 2016 to March 2021, at the Department of Vascular Surgery, Shaheed Mohtarma Benazir Bhutto (SMBB) Institute of Trauma, Karachi, Pakistan. The data were collected after approval from the institutional ethical review committee. Data were gathered from patients aged 17-70 years undergoing lower limb revascularization procedure following traumatic vascular injury, using a non-probability sampling technique.

Patients with crush injuries, non-salvageable limbs undergoing primary amputation, isolated deep femoral artery or crural arteries (non-axial arterial) injuries, and non-traumatic injuries like intravenous drug-induced or iatrogenic injuries were excluded. Patients with missing data were also excluded. Data were collected from the hospital database and patients' files and recorded on a predesigned proforma. Demographic details, mode of injury, limb involved, vessels involved, and association with fracture/dislocation, type of surgical procedure, fasciotomy, associated co-morbidities, and surgical outcomes were recorded. Primary outcome was recorded as amputation following revascularization procedure in the same index admission period.

The data were entered and analyzed using SPSS version 20.0 (IBM Corp., Armonk, NY, USA). Quantitative variables were presented as mean and standard deviation, while qualitative as percentages. Data were stratified for age, gender, mode of injury, vessel injured, fasciotomy, and the type of procedure and complication following surgical intervention. The post-stratification chi-square test was applied. A P-value <0.05 was considered as significant.

## Results

Total 56 patients meeting the eligibility criteria were included in the analysis. The male-to-female ratio was 4:1 and the mean age of 30.8 ± 9.29 years. Smoking was a more prevalent (32%) vascular risk factor, followed by hypertension (5.4%) and diabetes mellitus (3.6%). More than half of the patients did not have any co-morbidity. The mean time of injury to surgical intervention was 7.66 ± 1.69 hours. On exploration of the injury, the most common vascular injury was arterial laceration (50%), followed by arterial transaction (25%) and arterial thrombosis (17.9%), with a mean segment involvement of 1.14 ± 0.35. Among 56 patients, 27 (48.2%) patients were having concomitant open fractures, 21 (37.5%) patients were associated with closed fractures, while only eight (14.3%) patients were associated with dislocations. The superficial femoral artery and below-knee popliteal artery presented with the same frequency of 26.8%. The above-knee popliteal artery presented in 23.2%, deep femoral associated with superficial femoral in 16.1%, and the common femoral artery presented in about (7.1%) patients, as shown in Table 1.

**Table 1:** Descriptive analysis of data

Variables		Mean ± SD/Frequency
( n = 56)		
Age (years) (17-60 years)		30.82 ± 9.29
Gender	Male	46 (82.1%)
	Female	10 (17.9%)
Co-morbidities	Hypertension	3 (5.4%)
	Diabetes mellitus	2 (3.6%)
	Smoking	18 (32.1%)
	None	33 (58.9%)
Duration of injury (hours)		7.66 ± 1.69
Artery segments involved		1.14 ± 0.35
Type of Injury	Arterial laceration	28 (50%)
	Arterial transection	14 (25%)
	Arterial thrombosis	10 (17.9%)
	Arteriovenous fistula	4 (7.1%)
Associated Injuries	Fracture (closed)	21 (37.5%)
	Fracture (open)	27 (48.2%)
	Dislocation	8 (14.3%)
Artery injured	Common femoral artery	4 (7.1%)
	Deep femoral + superficial femoral artery	9 (16.1%)
	Superficial femoral artery	15 (26.8%)
	Popliteal artery (above knee)	13 (23.2%)
	Popliteal artery (below knee)	15 (26.8%)
Fasciotomy	Yes	38 (67.9%)
	No	18 (32.1%)
Amputation	Yes	18 (32.1%)
	No	38 (67.9%)
Cause of amputation	Graft thrombosis	8 (44.4%)
	Infection	5 (27.8%)
	Advanced necrosis	3 (16.7%)
	Others	2 (11.1%)
Time from presentation to amputation (hours)		55.98 ± 10.77

**Figure 1:**
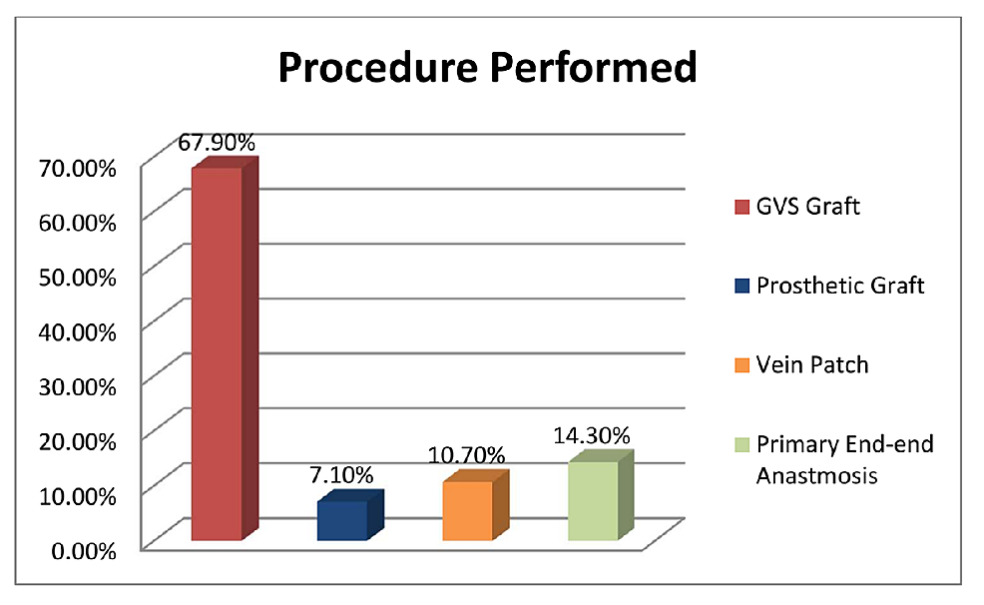
Primary revascularization procedure performed GVS, great saphenous vein

The great saphenous vein grafting in revascularization procedure was done in 38 patients (67.9%) while the use of prosthetic graft was limited (7%), as shown in Figure 1.

Post-revascularization fasciotomy was performed in about two-thirds of patients. Primary outcome (amputation) was seen in 18 (32.14%) patients. The average time of presentation of vascular injuries to delayed amputation was 55.98 ± 10.77 hours, and the most common reason for the failure of the revascularization procedure was thrombosis (41%). The least common reason was advanced necrosis (14%). The arterial segment involved, type of injury, and association with fracture (open/closed) or dislocation was significantly associated with the outcome (amputation), as shown in Table 2.

**Table 2:** Correlation of variables with outcome

Variables		Mean ± SD/Frequency	P-value
(n = 56)			
Type of injury (on exploration)	Arteriovenous fistula	4 (7.1%)	0.014
	Arterial laceration	28 (50%)	
	Arterial transection	14 (25%)	
	Arterial thrombosis	10 (17.9%)	
Associated injuries	Fracture (closed)	21 (37.5%)	0.034
	Fracture (open)	27 (48.2%)	
	Dislocation	8 (14.3%)	
Artery injured	Common femoral artery	4 (7.1%)	0.020
	Deep femoral + superficial femoral artery	9 (16.1%)	
	Superficial femoral artery	15 (26.8%)	
	Popliteal artery (above knee)	13 (23.2%)	
	Popliteal artery (below knee)	15 (26.8%)	

## Discussion

The devastating traumatic peripheral arterial injuries are currently managed sophisticatedly to improvise limb salvage with early vascular reconstruction [5,11]. The most important cause of poor outcomes following severe limb trauma is delayed decision-making and delayed revascularization [8]. Vascular injuries can impose risk to limb as well as life. Blunt trauma is associated with intimal hematoma and occlusion, whereas complete transection, arteriovenous fistula, and pseudoaneurysms are common in penetrating injuries [12].

The scarcity of level 1 trauma centers in rural and urban areas of Pakistan results in higher amputation rates after peripheral vascular injuries. Limited resources and expertise coupled with delay in diagnosis and referral to appropriate trauma center lead to poor outcomes of these injuries. Our institute (SMBB Institute of Trauma), being the only public sector hospital providing vascular surgery services to most of the province's population, deals with a majority of vascular injuries. Out of 56 patients, about one-third of the patients ended up with amputation while two-thirds had successful revascularization, with a majority of these amputations occurring within three days of revascularization. Majority of patients were of young age (mean age 30.82 ± 9.29) with a male-to-female ratio of 4:1. In our study, the most frequent injured arterial segment was popliteal artery followed by SFA. These findings are comparable to those reported by other studies [13-15]; however, in a few western studies, the popliteal artery injuries were lower, i.e. <0.25% [16]. Most of the study population was young, male, and having no comorbid conditions. 

The mean duration of injury to the revascularization procedure was 7.66 ± 1.69 hours. Although, prolonged ischemia (>6 hours ) is associated with four times higher risk of secondary amputation. However, there is evidence of successful limb salvage following arterial injury in literature even after 24 hours of injury [17]. We assessed vascular injuries, the number of segments involved, and the availability of collateral by using readily available color Doppler ultrasound as it has 95% sensitivity and 99% specificity [18]. Vascular trauma is mostly associated with some bony and soft tissue injuries. This study found open fractures (48%), close fractures (37%), and dislocation (14%) associated with arterial injuries, significantly affecting the outcome (P = 0.034). On exploration, arterial laceration was found to be most prevalent, followed by arterial transaction and thrombosis with a frequency of 50%, 25%, and 18%, respectively. The most common procedure performed was autologous great saphenous venous graft. These findings were consistent with similar results in the literature [15,19]. Fasciotomy was performed in 38 patients out of 56 patients with impending compartment syndrome or prophylactically after arterial repair. Fasciotomy is considered a standardized protocol following reconstruction as a preventive measure for compartment syndrome. However, a cohort study by Kauvar et al. concludes that fasciotomy was not associated with limb salvage but complications [20].

The most common reason for failed revascularization was arterial thrombosis and infection in about two-thirds of patients in our studied population. Perkins et al. [21] pointed out that the anatomical site of an arterial injury is an essential prognostic risk factor of amputation. Similar to Perkins et al., we found that anatomical site and also the type of arterial injury and associated injuries have greatly impacted the prognosis of revascularization as a limb salvage procedure.

This study carries the limitations of a retrospective design, with a significant number of patients excluded due to missing data on variables. Also, this is a single-center review with a limited number of patients. Besides the above, this study does not include patients who underwent a second revascularization procedure, complications of salvage procedures, and the functional outcome of limb salvage procedure with follow-up.

## Conclusions

Type of arterial injury, arterial segment involved, and the associated bony injuries are associated with post-revascularization amputations in lower limb arterial injuries. However, multicenter prospective studies are recommended to overcome the limitations of this study.
